# Evaluating the Antioxidant Properties of Unifloral Honey (*Apis mellifera* L.) from Ethiopia

**DOI:** 10.1155/2023/7664957

**Published:** 2023-07-15

**Authors:** Ofijan Tesfaye

**Affiliations:** Oromia Agricultural Research Institute, Haro Sebu Agricultural Research Center, Oromia, Ethiopia

## Abstract

The antioxidant properties of natural honey primarily rely on the floral origin from which nectar is collected by bees. Thus, the current activity evaluated the antioxidant properties of honey based on its floral type. The honey floral origin was verified by the melissopalynological technique. Antioxidant properties were determined by using standard procedures and analyzed by SAS. Six unifloral honey types with their harvesting month were identified. Accordingly, *Guizotia* (74% of pollen frequency), *Coffea arabica* (68%), *Vernonia* (90%), *Croton macrostachyus* (64%), *Schefflera abyssinica* (100%), and *Eucalyptus* (100%) were cropped in November, February, February, May, April, and June separately. Statistically, a variation (*p* < 0.05) in antioxidant parameters was displayed between unifloral honeys. *Vernonia* honey exhibited the maximum total phenol (77.2 ± 0.7), total flavonoid (65.0 ± 3.8), and total antioxidant content (65.4 ± 0.3). On the other hand, *S. abyssinica* honey recorded the least total phenol content (24.1 ± 0.4), total flavonoid content (18.6 ± 2.7), and total antioxidant content (5.6 ± 0.5). Statistical analysis showed a positive correlation between all the tested antioxidant parameters. Thus, the current study indicated that all the tested Ethiopian unifloral honey had good sources of antioxidants with the most *Vernonia* honey followed by C*. macrostachyus* whereas *S. abyssinica* honey had the least followed by *Eucalyptus*.

## 1. Introduction

Free radicals are formed in our body during a chemical reaction and lead to damage our cell. It's inhibited by antioxidants produced during our daily natural food. As indicated by Wu and Cederbaum [1], free radicals are too sensitive as they form a bond with other substances, particles, or individual electrons to produce a constant compound and reactive oxygen species take place. This causes many diseases like the formation of cancer, infection, getting old, pathogenesis and development of diabetes [2], circulatory illness, immunity failure, degenerative diseases of the nervous system , heart and lung diseases, and eye problem [3]. Eating natural antioxidants from natural products like honey is active in the hindrance of prolonged illnesses that have amplified in current time [2, 4].

Honey is a normal nutritive antioxidant whose composition is accountable for the redox properties, namely, flavonoids, phenols, enzymes, vitamins, and minerals [5]. Honey is synthesized by bees either from a single plant (its honey is called unifloral honey) or multiple plant species (its honey is known to be multifloral honey), and honey antioxidant activities are determined by its plant and geographical origin, humidity, temperature, climate, and environment condition [6]. The unifloral and multifloral honey sample is authenticated by the melissopalynological method, and taxon of the pollen is typically used to point out the plant nectar origin collected by bees to synthesize honey [7]. Ethiopia has more than ten million colonies, and above eight hundred recognized bee plant [8]. Moreover, the suitability of geographical position, plant diversity, and climatic conditions in Ethiopia makes the topmost honey producer in Africa and tenth from the worldwide [9]. The goal of this work was to screen the antioxidant properties of unifloral honey harvested in Ethiopia.

## 2. Materials and Methods

### 2.1. Sample Assortment

It was collected as of different areas of the country based on the accessibility of unifloral honey, and their latitude and longitude are indicated in Table 1. Accordingly, C. arabica and C. macrostachyus honey from Haro Sebu, Guizotia honey from Nejo, Vernonia honey from Gedo, Eucalyptus honey from Holota, and S. abyssinica honey from Bore were collected from farmer beekeepers' apiary site based on their honey harvesting calendar. An overall 30 kg (sample size) of honey trials for each unifloral honey type, from 30 dissimilar apicultures (around 1 kg per farmer) of the study site, has taken. Then after, the representative trials were transported to the University of Addis Ababa, Lab. of Food Science, using sterilized beaker. At the time of cropping, visual remarking of hive environs was done besides group discussion with skilled apicultures of every site to acquire the nectar origin of the samples and flowering bee plants available in the site.

### 2.2. Floral Source Analysis

The technique of Louveaux et al. [7] was used. Hence, 10 g of honey was added in 20 mL of sterile distilled water. The honey solution was centrifuged at 3800 rpm for 10 minutes, and the supernatant was poured out. Then, 20 mL of distilled water was again added to completely dissolve the remaining sugar crystals and centrifuged at 3800 rpm again for 5 minutes, and the supernatant was removed completely. The sediment was spread evenly using a sterile micro spatula on the microscope slide, and the sample was dried for a while. Thereafter, one drop of glycerin jelly was added to the coverslip, and the pollen grains were identified using a pollen atlas [8] which was prepared for plant identification from honey sample. Moreover, pollen morphology types were verified by comparison with reference slides of pollen assorted directly from the live flower plants in the study area. Then after, bee plant species from honey sample was identified; their contribution to bees (pollen, nectar, or both) and life form was known at field and different literatures [7, 8]. The percentage of pollen types in each honey sample was calculated based on the total number of different types of pollen grains counted in each sample. Accordingly, if >45% of counted pollen grain was from specific plant species, it was categorized under predominant pollen (monofloral honey); if 16-45%, secondary pollen; if 3-15%, important minor pollen; and if <3%, minor pollen, while honey sample with no predominant pollen was used as mixed honey type [7]. The pollen count was determined under a light microscope (Swift Instrument International, serial number 8750038, Japan, high power 400x) linked to a computer.

### 2.3. Antioxidant Properties of Honey

#### 2.3.1. Total Phenolic Contents

**Figure 1:**
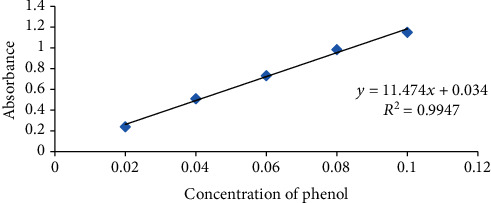
Calibration curve for phenol.

The phenol content of unifloral honey was assessed by the Folin-Ciocalteu method [5]. Honey stock solution was formed by dissolving 2 g of the honey in 25 mL of distilled water and strained by Whatman no. 1. Then, 0.5 mL aliquot from stock solution was mixed with 2.5 mL of 0.2 N Folin-Ciocalteu reagent and stored for 5 min. A 2 mL of 75 g/L sodium carbonate solution was added to the solution and incubated for 2 h at 25°C. Finally, the absorbance of the mixture was calculated at 765 nm using UV (PerkinElmer Lambda 950 UV/VIS/NIR Spectrophotometer). A standard chemical taken to create a calibration curve was gallic acid (0-200 mg/L) as a control. Lastly, composition of total phenol was stated as milligrams of gallic acid per one hundred grams of honey from an average result of triplicate data. The calibration formula (*y* = 11.474*x* + 0.034; *R*^2^ = 0.9947) was derived from the calibration curve (Figure 1).

#### 2.3.2. Total Flavonoid Content (TPC)

**Figure 2:**
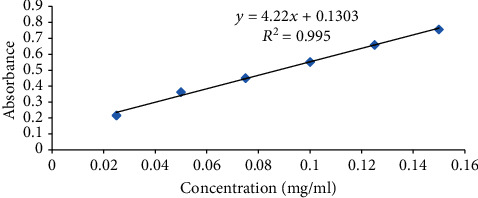
Calibration curve for flavonoid.

The procedure by Chua et al. [5] was used. For this, a mixture of 5 g honey in 50 mL distilled water was used as a honey stock solution, and out of this, 5 mL was dropped in 5 mL of 2% AlCl_3_ solution and incubated for 10 minutes. Then, its absorbance was read at 415 nm by spectrophotometer. Then, for calibration curve formulation, a standard chemical which is quercetin (0-200 mg/L) as a control was chosen. This procedure was triplicated and stated as milligrams of quercetin per 100 grams of honey from the averaged result of triplicate data. The calibration equation (*y* = 4.22*x* + 0.1303; *R*^2^ = 0.995) was derived from the calibration curve (Figure 2).

#### 2.3.3. The Antioxidant Composition

**Figure 3:**
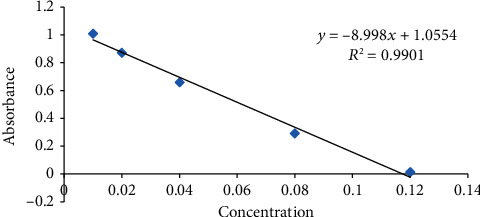
Calibration curve for ascorbic acid.

It was founded by calculating the ascorbic acid equal antioxidant capacity (AAEAC) using usual procedures [10]. For this, 0.5 milligrams of DPPH was dissolved in twenty-five millilitres of methanol to get DPPH solution. As well, 30 mg of honey was mixed in millilitre methanol. Then after, 0.75 mL honey solution was mixed in 1.5 mL of DPPH solution. After the solution was incubated at room temperature for 15 min, its absorbance was measured at 517 nm. A mixture of 0.75 mL of a methanolic honey solution with 1.5 mL of methanol was used as a blank. A calibration curve was produced from a standard chemical: ascorbic acid (0-200 mg/L) (control). The procedure was triplicated and stated as milligrams of ascorbic acid per 100 grams of honey. The calibration equation (*y* = −8.998*x* + 1.0554; *R*^2^ = 0.9901) was derived from the calibration curve (Figure 3).

### 2.4. Statistical Analysis

Average and standard deviations were calculated using SAS Software. Significant variation between unifloral honeys was determined using one-way ANOVA. Antioxidant parameters were used for mean separation by least significant difference.

## 3. Results and Discussion

### 3.1. Floral Source Result

**Figure 4:**
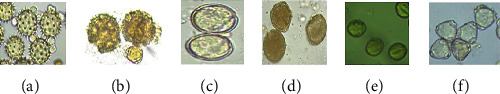
Pollen pictures of bee floras that provide unifloral honey in Ethiopia: (a) *Guizotia* spp. (predominant pollen, 74.10%), (b) *Vernonia* spp. (predominant pollen, 90.00%), (c) *C. arabica* (predominant pollen, 68.01%), (d) *C. macrostachyus* (predominant pollen, 64.42%), (e) *S. abyssinica* (predominant pollen, 100%), and (f) *Eucalyptus* spp. (predominant pollen, 100%).

**Figure 5:**
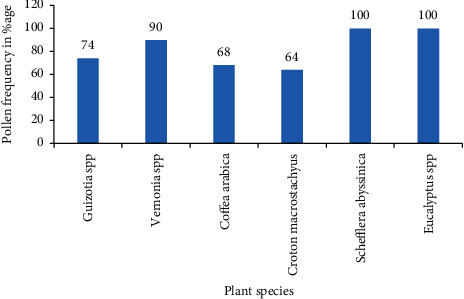
Percentage of pollen grain frequency of unifloral honey types.

All floral honey source with their characteristics such as harvesting time, life form, and resource released for bees and pollen frequency category is depicted in Table 2. Pollen pictures of bee floras that provide unifloral honey are illustrated in Figure 4. All the time, honey sample includes various pollen grains which provide a good fingerprint of the geographical and botanical origin where the nectar is collected from [11]. After the pollen grain was counted and the percentage calculated, all honey types were categorized as unifloral honey since their pollen frequency was greater than 45%. Accordingly, there are six unifloral honey types, namely, (1) *Guizotia* honey from the Nedjo area harvested through November, (2) *C. arabica* honey from the Haro Sebu area harvested through February, (3) *Vernonia* honey from Gedo harvested through February, (4) *C. macrostachyus* from Haro Sebu area harvested through May, (5) *S. abyssinica* honey from Bore harvested through April, and (6) *Eucalyptus* honey from Holota harvested through June. The percentage pollen frequency from *Guizotia*, *C. arabica*, *Vernonia*, *C. macrostachyus*, *S. abyssinica*, and *Eucalyptus* honey samples was 74, 68, 90, 64, 100, and 100, respectively (Figure 5).

Similarly, in Ethiopia, *Guizotia*, *Vernonia*, *C. arabica*, and *S. abyssinica* honeys are harvested from November to December, February, February through March, and April through May [12], respectively. Not all flowering plants equally contribute to the bees. Nectar quality (sugar content), potentiality, and abundance of the plant in a given area contribute to cropping a unifloral honey (predominant pollen source). From this study, secondary pollen contributor (*Vernonia* plant) has occurred in *C. arabica* honey. The study area is well known in coffee production and when flowered is abundantly found and stays for a short period of flowering (less than 10 days). This is concurrent with [13] who observed an overlapping of the *Vernonia* plant flowering period with *C. arabica* from honey samples harvested from February through March.

### 3.2. Antioxidant Properties

#### 3.2.1. Total Phenolic Content (TPC)

The present study screened honey samples between plant sources, and a very significant disparity (*p* < 0.05) of TPC was obtained in all the tested honey types. TPC is articulated as milligrams of gallic acid per 100 g of honey. It ranged from a mean of 24.1 ± 0.4 by *S. abyssinica* to 77.2 ± 0.7 by *Vernonia* (Table 3). The current result is that the indication of *Vernonia* honey has a high antioxidant content followed by *C. macrostachyus* while *S. abyssinica* honey is a weak antioxidant compound content.

The total phenol substance in honey is examined by TPC which is a fast and simple technique [5]. Moreover, Al et al. [14] demonstrated that TPC was an adequate parameter for an overall phenol approximation in honey and is directly interconnected to the antioxidant action of honey. Furthermore, number of phenols found in the honey sample highly relies on the floral source from which honey is synthesized and is one of the greatest vital classes of substances existing in honey [15].

The TPC of the current study is less than that of Malaysian honey (110.4-196.5 milligram GAE in hundred gram honey) [5] and Sudanese honey (201.1 ± 2.5 milligram GAE in hundred gram honey) [16]. However, it is found higher than in Germany (4.6 mg/100 g honey) [17] and Slovenia (4.48 mg GAE/100 g honey) [18]. This finding was found within the reported ranges of Northeast Brazilian honey (27.0 to 92.7 mg GAE/100 g) [19] and Ethiopian honey (233.3 ± 24.0 mg GAE/kg to 693.3 ± 26.8 mg GAE/kg) [20]. Nevertheless, as in my study, comparable and higher TPC was investigated by *V. amygdalina* (693.3 ± 26.8 mg GAE/kg) followed by *C. macrostachyus* (574.2 ± 40.8 mg GAE/kg) [20] which is an observer of phenolic content in honey is an indication of its floral source. On the other side, the difference in TPC might be due to floral and environmental origin, method of honey harvesting, duration of honey storage, laboratory procedure, and chemical and reagents used during the laboratory analysis.

#### 3.2.2. Total Flavonoid Content (TFC)

It is stated as milligrams of quercetin per 100 g of honey. The presence of flavonoid significantly contributes to the overall antioxidant action of honey, hence take positive properties on human healthiness [21, 22]. An important disparity (*p* < 0.0001) was cropped among all honey types. As with the TPC, the lowest and highest TFC results of the current study ranged from 18.6 ± 2.7 by *S. abyssinica* honey to 65.0 ± 3.8 by *Vernonia* honey (Table 3). Comparably, [23, 24] demonstrated that honey samples with higher phenolic substance will similarly yield high flavonoid. The flavonoid from the current finding was greater than honey from Turkey (1.1 to 9.2 milligram QE/100 g) as of *A. mellifera* [25] and marketable honey from Portugal (1.7 ± 0.8 milligram QE/100 g in citrus) from *A. mellifera* [26].

#### 3.2.3. Antioxidant Content (AC)

AC of the current study is defined as milligrams of ascorbic acid equal in hundred grams of the sample. As those of TPC and TFC, *Vernonia* honey demonstrated the highest AC (65.4 ± 0.3) followed by *C. macrostachyus* (27.4 ± 1.1). However, statistically similar (*p* > 0.05) and less AC was obtained by *Guizotia*(9.0 ± 4.5), *C. arabica* (6.4 ± 1.4), *Eucalyptus* spp. (5.9 ± 0.1), and *S. abyssinica* honey (5.6 ± 0.5) (Table 3). The surprisingly highest result was obtained from *Vernonia* honey in TPC, TFC, and AC.

The AC of the current result was comparable to those from Malaysian honey that recorded an average of 14.23 to 26.64 mg AEAC/100 g [27], Pakistani natural honey (8.30–22.10 mg AEAC/100 g [28]), multifloral Burkina Fasan honey (10.20–37.87 mg AEAC/100 g [29]), and Czech honey from 14.15 to 40.71 mg AEAC/100 g [30] while less than Manuka honey (84.47 mg/100 g) [27].

Comparably, Adgaba et al. [20] have recorded higher antioxidant capacities by *V. amygdalina* and *C. macrostachyus* while multifloral and *Guizotia scabra* honey produced relatively lower. The disparities in phytochemicals of the particular honey floras and environmental origin could bring variation in antioxidant properties. Similarly, differences in antioxidant activities of different honeys based on floral and environmental origin and seasonal factors are well testified [31, 32].

#### 3.2.4. Correlation between TPC, TFC, and AC

The correlation matrix is depicted in Table 4. A strong and significant positive correlation was observed among TPC and TFC (*r* = 0.80, *p* < 0.0001) and TPC and AC (*r* = 0.70, *p* < 0.01). Besides, TFC and AC showed a moderate and important positive association (*r* = 0.69, *p* < 0.001). From the current result, the antioxidant properties of any honey type are determined by its phenolic, flavonoid, and antioxidant contents. As the phenolic content of a given honey type increases, then its flavonoid content is also increased and their aggregation could exhibit high antioxidant compounds of honey. The beneficial effect of honey on the health of humans is determined by its phenolic and flavonoid contents. The total phenolic substance in honey is sensitive to polyphenol entities, ascorbic acid, and vitamin E [33].

Comparable correlation with the current study was obtained between TPC and TFC (*r* = 0.776) and TFC and AC (0.730) [24]. In contradiction with this finding, there is no correlation between TPC and AC (0.165) from Algeria [24] and Czech [30]. A. mellifera honey was observed. However, a highly positive correlation between AEAC and TPC (*r* = 0.968) from Malaysian raw honey [27] and a positive association between flavonoid, phenolic, and antioxidant activities in Brazilian honey [34] were exhibited which are similar with this result.

## 4. Conclusion

This study showed that the country has a potential for cropping different brands of honey owing to the availability of dominant honey plant diversity in a different environments. No study was carried out on the harvesting period and antioxidant properties of honey based on floral origin. Unifloral honey types, namely, *Guizotia*, *C. arabica*, *Vernonia*, *C. macrostachyus*, *S. abyssinica*, and *Eucalyptus*, could be cropped in November, February, February, May, April, and June, respectively, where they abundantly occurred and are major honey plants in Ethiopia. Based on the current result, all the tested honey types exhibited good antioxidant properties with the highest TPC, TFC, and AC by *Vernonia* followed by *C. macrostachyus* while *S. abyssinica* was the least followed by Eucalyptus honey. Moreover, TPC, TFC, and AC had a strong positive correlation and are important parameters for the antioxidant constituents of the honey sample.

## Figures and Tables

**Table 1:** Latitude and longitude of each locations of unifloral honey sampled.

No.	Location	Latitude	Longitude
1	Haro Sebu	8° 44′ 59.99^″^ N	35° 19′ 60^″^ E
2	Nedjo	10° 49′ 21^″^ N	35° 14′ 19^″^ E
3	Gedo	9° 01′ 0^″^ N	37° 27′ 0^″^ E
4	Holota	9° 4′ 0^″^ N	38° 30′ 0^″^ E
5	Bore	6° 21′ 35^″^ N	38° 37′ 20^″^ E
6	Haro Sebu	8° 44′ 59.99^″^ N	35° 19′ 60^″^ E

**Table 2:** Honey plants identified from each of the honey types.

Site	Harvesting time	Scientific name	Family name	Vernacular name (Afaan Oromo)	Life form	Resources for bees	Pollen grain counted (%)	Frequency class	Honey type
Nedjo	November	*Guizotia* spp.	Asteraceae	Tufo/nuugii	Herb	P & N	74.1	PP	Monofloral (*Guizotia* spp.) honey
		*Bidens* spp.	Asteraceae	Maxxannee	Herb	P & N	5.9	IMP	
		*Trifolium* spp.	Fabaceae	Siddisa	Herb	P & N	4.1	IMP	
		*Eucalyptus* spp.	Myrtaceae	Baargamoo	Tree	P & N	4.1	IMP	
		*Grass* type	Poaceae	Gosamargaa	Herb	P & N	3.0	IMP	
		*Plantago lanceolata*	Plantaginaceae	Qorxobbii	Herb	P	2.6	MP	
		*Sesamum indicum*	Pedaliaceae	Saalixa	Herb	P & N	1.8	MP	
		*Allophylus abyssinicus*	Sapindaceae	Sarara	Tree	P & N	1.6	MP	
		*Vicia faba*	Papilionaceae	Baaqelaa	Herb	P & N	1.5	MP	
		*Lepidium sativum*	Brassicaceae	Feexoo	Herb	P & N	1.1	MP	
		*Brassica carinata*	Brassicaceae	Raafuu	Herb	P & N	0.2	MP	
		*Satureja paradoxa*	Lamiaceae	Xinaaddama	Herb	P & N	0.04	MP	

Haro Sebu	February	*Coffea arabica*	Rubiaceae	Buna	Shrub	P & N	68.0	PP	Monofloral (C. arabica) honey
		*Vernonia* spp.	Asteraceae	Gosaeebichaa	Shrub	P & N	17.46	SP	
		*Terminalia* spp.	Combretaceae	Birdheessa/dabaqqa	Tree	P & N	5.16	IMP	
		*Guizotia* spp.	Asteraceae	Tuufoo/hadaa	Herb	P & N	3.67	IMP	
		*Apodytes dimidiata*	Icacinaceae	Qumbaala	Tree	P & N	2.15	MP	
		*Bidens* spp.	Asteraceae	Keelloo	Herb	P & N	1.57	MP	
		*Hypoestes triflora*	Acanthaceae	Darguu	Herb	P & N	1.22	MP	
		*Plantago lanceolate*	Plantaginaceae	Qorxobbii	Herb	P & N	0.60	MP	
		*Galiniera saxifraga*	Rubiaceae	Mixoo	Shrub	P & N	0.53	MP	
		*Justicia schimperiana*	Acanthaceae	Dhummuuggaa	Shrub	P & N	0.35	MP	
		*Bersama abyssinica*	Melianthaceae	Lolchiisaa	Tree	P & N	0.35	MP	
		*Rumex nervosus*	Polygonaceae	Dhangaggoo	Shrub	P & N	0.15	MP	
		*Euphorbia ampliphylla*	Euphorbiaceae	Adaamii	Tree	P & N	0.13	MP	

Haro Sebu	May	*Croton macrostachyus*	Euphorbiaceae	Bakkanniisa	Tree	P & N	64.42	PP	Monofloral (C. macrostachyus) honey
		*Syzygium guineense*	Myrtaceae	Baddeessaa	Tree	P & N	14.03	IMP	
		*Eucalyptus* spp.	Myrtaceae	Baargamoo	Tree	P & N	12.57	IMP	
		*Acacia* spp.	Myrtaceae	Laaftoo	Tree	P & N	7.81	IMP	
		*Justicia schimperiana*	Acanthaceae	Dhummuuggaa	Shrub	P & N	0.55	MP	
		*Rumex nervosus*	Polygonaceae	Dhangaggoo	Shrub	P & N	0.38	MP	
		*Coffea arabica*	Rubiaceae	Buna	Shrub	P & N	0.27	MP	
		*Guizotia* spp.	Asteraceae	Tuufoo	Herb	P & N	0.16	MP	
		*Bidens* spp.	Asteraceae	Keelloo/Maxxannee	Herb	P & N	0.03	MP	

Gedo	February	*Vernonia* spp.	Asteraceae	Eebicha/Reejjii	Shrubs	P & N	90	PP	Vernonia (monofloral) honey
		*Eucalyptus camaldulensis*	Myrtaceae	Baargamoo	Tree	P & N	3.02	IMP	
		*Hypoestes triflora*	Acanthaceae	Darguu	Herb	P & N	2.6	MP	
		*Apodytes dimidiata*	Icacinaceae	Qumbaala	Tree	P & N	1.4	MP	
		*Plantago lanceolate*	Plantaginaceae	Qorxobbii	Herb	P & N	1.3	MP	
		*Cardus nyassanus*	Asteraceae	Qoreeharree	Herb	P & N	1.04	MP	
		*Rumex* spp.	Polygonaceae	Dhangaggoo	Shrub	P & N	1.04	MP	
		*Guizotia* spp.	Asteraceae	Tuufoo	Herb	P & N	0.5	MP	

Bore	April	*Schefflera abyssinica*	Araliaceae	Gatamaa	Tree	P & N	100	PP	*S. abyssinica* (monofloral) honey

Holota	June	*Eucalyptus* spp.	Myrtaceae	Baargamoo	Tree	P & N	100	PP	*Eucalyptus* (monofloral) honey

PP = predominant pollen (if >45% counted pollen grain was from specific plant species); SP = secondary pollen (16%-45%); IMP = important minor pollen (3%-15%); MP = minor pollen (<3%); P & N = pollen and nectar.

**Table 3:** Antioxidant properties between the tested unifloral types.

Parameters	Unifloral types (*average* ± *standard* *deviation*)						*X*	LSD	P-V	CV
	*Vernonia* honey	*C. macrostachyus* honey	*Guizotia* honey	*C. arabica* honey	*Eucalyptus* honey	*S. abyssinica* honey				
TPC (mg GAE/100 g of honey)	77.2 ± 0.7^a^	70.3 ± 1.6^b^	57.6 ± 0.3^c^	47.9 ± 2.2^c^	26.3 ± 1.1^e^	24.1 ± 0.4^f^	49.2	2.1	<0.0001	2.4
TFC (mg QE/100 g of honey)	65.0 ± 3.8^a^	53.4 ± 0.5^b^	31.5 ± 0.7^d^	39.7 ± 2.2^d^	26.2 ± 0.2^e^	18.6 ± 2.7^f^	40.4	3.8	<0.0001	5.4
AC (mg AAE/100 g of honey)	65.4 ± 0.3^a^	27.4 ± 1.1^b^	9.0 ± 4.5^c^	6.4 ± 1.4^c^	5.9 ± 0.1^c^	5.6 ± 0.5^c^	19.9	3.5	<0.0001	10

Different superscripts in a row vary statistically at a 1% probability level. Note: mg GAE/100 g of honey = milligrams of gallic acid equivalent in 100 g of honey; mg QE/100 g of honey = milligrams of quercetin equivalent in 100 g of sample; mg AAE/100 g of honey = milligrams of ascorbic acid equal in hundred grams of honey; *X* = mean; LSD = least significant difference; P-V = *p* value; CV = coefficient of variation in % age.

**Table 4:** Pearson correlation between TPC, TFC, and AC.

	TPC	TFC	AC
TPC	1		
TFC	0.80375^∗^	1	
AC	0.8737^∗^	0.77178^∗^	1

^∗^ is significant at a 1% probability.

## Data Availability

The datasets used and/or analyzed during the current study are available upon reasonable request from the relevant author.
